# Advances in understanding – genetic basis of intellectual disability

**DOI:** 10.12688/f1000research.7134.1

**Published:** 2016-04-07

**Authors:** Pietro Chiurazzi, Filomena Pirozzi

**Affiliations:** 1 Institute of Genomic Medicine, Catholic University School of Medicine, Rome, Italy; 2 Department of Genetics and Genome Sciences, Case Western Reserve University School of Medicine, Cleveland, Ohio, USA

## Abstract

Intellectual disability is the most common developmental disorder characterized by a congenital limitation in intellectual functioning and adaptive behavior. It often co-occurs with other mental conditions like attention deficit/hyperactivity disorder and autism spectrum disorder, and can be part of a malformation syndrome that affects other organs. Considering the heterogeneity of its causes (environmental and genetic), its frequency worldwide varies greatly. This review focuses on known genes underlying (syndromic and non-syndromic) intellectual disability, it provides a succinct analysis of their Gene Ontology, and it suggests the use of transcriptional profiling for the prioritization of candidate genes.

## Introduction

The advances in scientific technology related to gene sequencing and discovery in recent years, such as high-throughput whole genome sequencing (WGS) and single-cell sequencing, have led to an increasing number of studies aimed at finding new causative genes for human diseases.

**Figure 1.**
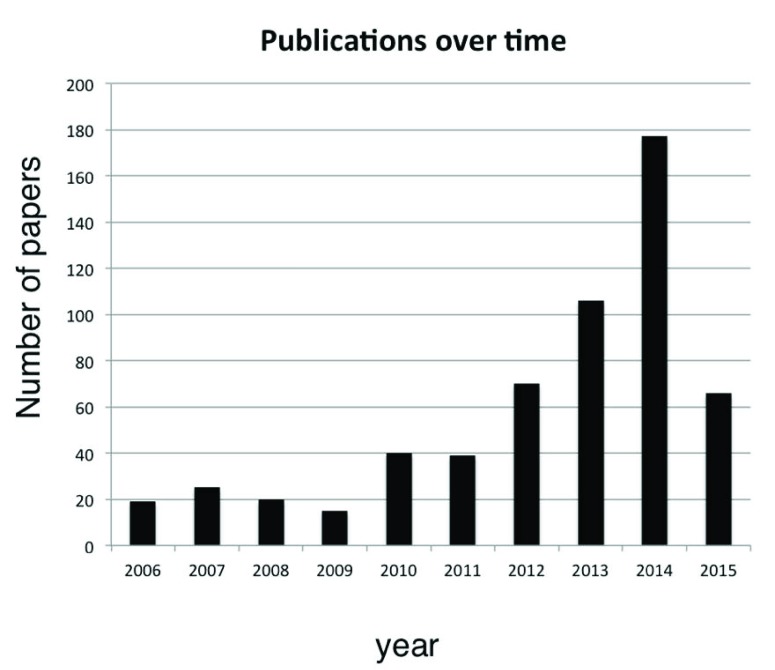
Bar graph illustrating the number of publications per year from 2007 to 2015 obtained with the PubMed search “(intellectual disability OR mental retardation) AND (next-generation sequencing OR exome sequencing)”.

Owing to the heterogeneity of clinical features and causative factors (both genetic and environmental), characterization of intellectual disability (ID) has benefited from these advances, as shown by the significant increase of publications ( Figure 1). As defined by the *Diagnostic and Statistical Manual of Mental Disorders, Fifth Edition* (DSM-5), ID is characterized by significant limitations in intellectual functioning and adaptive behavior, which include conceptual, social, and practical skills, arising “prior to age 18” (but it would be fair to say “with a prenatal origin”). The disorder is considered chronic and often co-occurs with other mental conditions like depression, attention deficit/hyperactivity disorder, and autism spectrum disorder (ASD). Furthermore, ID is often part of a malformation syndrome that affects other organs and their functions.

ID is the most common developmental disorder; however, given the heterogeneity of its causes, estimates of its frequency worldwide are highly variable (reviewed by 1). The prevalence of ID also varies depending on the age of patients, as illustrated by two Australian surveys in which prevalence was 3.3/1000 if the age range of 20 to 50 years was considered ^2^ but increased to 14.3/1000 if the age range was lowered to 6 to 15 years ^3^.

The purposes of this review are to update the list of known genes related to ID and to provide a brief bioinformatic analysis of their Gene Ontology (GO). Eventually, we propose the use of a relative expression ratio (“Brain ratio”) to prioritize new candidate genes for ID.

## Nomenclature: mental retardation versus intellectual disability

Changes in nomenclature (i.e. how we name things and concepts) are particularly delicate in science, since consistency in terminology allows more precise communication ^4^. As discussed elsewhere ^5^, “mental retardation” has recently been substituted by the new term ID, which in our opinion is less accurate since it does not refer to the developmental nature of the disease and it does not reflect the progress of mental acquisitions that could nonetheless be achieved but at a slower pace. That said, we should remember that ID is not the only term employed to indicate delayed acquisition of psychomotor milestones. In fact, “developmental delay” is the second most common term found in the Clinical Synopsis of OMIM (Online Mendelian Inheritance in Man) (after “mental retardation”) and is widely used by pediatricians ^6^. Other more complex terms that have been proposed, such as “intellectual developmental disorder”, “neurodevelopmental disorder”, or “developmental cognitive impairment” ^7^, though certainly more accurate than ID, have not gained in popularity. However, all of these terms refer to the slower acquisition of psychomotor milestones, resulting in a significant impairment of cognitive functions (a) and adaptive behavior (b), obviously with an early onset (c), compared with peers. Cognitive abilities can be measured by using a panoply of psychological tests, including the Wechsler Intelligence Scale for Children, that have as the output a numerical value known as “intelligence quotient” (IQ).

It is worth remembering that the term “pervasive developmental disorder” (PDD) is often used by psychologists and psychiatrists to refer to a group of conditions characterized by altered development of multiple basic functions, including socialization and communication. In May 2013, the DSM-5 was released and the term PDD was abandoned and substituted by ASDs. Finally, the term “learning disability” is usually reserved for specific impairments, like dyslexia and dyscalculia, that are associated with a child’s academic underperformance but not with a lower IQ.

Nomenclature also reflects social trends and sensibilities that vary with time and according to the different cultural context. Social perception has become a decisive factor in changing nomenclature: the term “mental retardation” is not considered politically correct any longer because of the pejorative term “retard” that is used to stigmatize affected individuals ^4^. The community of parents and patients has indeed shown strong disagreement with the term “mental retardation”, leading to “Rosa’s law”, signed by President Obama on October 5, 2010. The new bill requires the federal government to replace the term “mental retardation” with ID in every context. Therefore, we will use the term ID in this article to refer to “mental retardation” from now on.

## Environmental and genetic causes of intellectual disability

**Figure 2.**
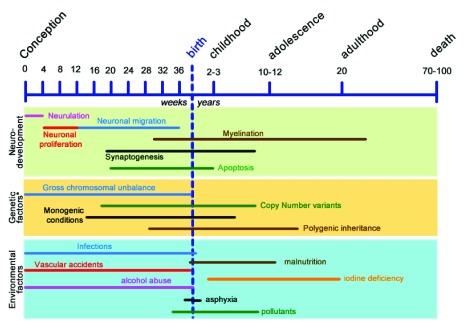
Schematic representation of neurodevelopmental stages, related to genetic and environmental factors and their time window. *For genetic factors, the onset of symptoms or time of detection is shown. Modified from 79.

ID can be caused by a variety of environmental and genetic causes, often combined with each other ^8– 12^. As illustrated in Figure 2, most of these causes exert their effects already during prenatal life. As indicated in table 1 of Chiurazzi and Oostra ^13^, the severity of the clinical presentation is loosely correlated with the causal factor, and gross chromosomal imbalances, perinatal asphyxia, prenatal infections, or vascular accidents are related to the most severe cases. Variable (and dose-related) effects result from maternal exposure to toxic substances during pregnancy (e.g. environmental chemicals, use of drugs, and alcohol abuse), maternal conditions such as diabetes or phenylketonuria, and premature birth. Common (but preventable) environmental causes of ID are iodine deficiency and malnutrition (of both mother and child), affecting millions of people in “developing countries”. The frequency of these various factors varies greatly among different countries and depends on (maternal) lifestyle as well as health-care quality.

Mendelian causes of ID result in highly variable phenotypes ranging from mild (IQ of 55–70) to moderate (IQ of 40–55), severe (IQ of 25–40), and profound (IQ of less than 25), depending on the gene or genes involved, the effects of the mutation (dosage changes, loss-of-function mutation, and gain-of-function mutations), and the function(s) of the altered protein(s).

Clinically, it is useful to distinguish syndromic from nonsyndromic forms of ID, depending on the involvement of other organs and the presence (or absence) of malformations or a typical (facial) *gestalt* (or both). However, it is not uncommon to observe that some mutations in a given gene cause a syndrome but that other mutations in the same gene lead to nonsyndromic or “pure” forms of ID. Comorbidity with autism, epilepsy, and neuromuscular deficits (e.g. ataxia, spastic paraplegia, sensory/motor neuropathy, and muscular dystrophy) is common for nonsyndromic ID.

Development of a functional brain depends on a precise and complex sequence of neuronal and glial cell proliferation, migration, and maturation. Some ID syndromes are associated with gross brain malformations (e.g. holoprosencephaly, schizencephaly, porencephaly, hydrocephalus, agenesis of corpus callosum, and cerebellar hypoplasia) or with neuronal migration disorders (e.g. lissencephaly, micropolygyria, double cortex, and ventricular nodular heterotopia) that can be assessed by neuroimaging techniques. However, even in the presence of a morphologically normal brain, neuronal connectivity could be altered by a dysfunction of the glia (e.g. disorders of myelination) or neuronal crosstalk might be altered at the synaptic level, either because of a reduced number of mature dendritic spines or because of inefficient (or excessive) synaptic transmission ^14^. Finally, even if both neurons and glial cells are well positioned, connected, and working, they could be damaged by toxic compounds accumulating in metabolic disorders (toxic neurodegeneration). A careful clinical evaluation of the patient(s), including reconstruction of personal and family history, possibly integrated by neuroimaging or neurophysiological tests or both, may provide essential clues to reach a diagnosis and identify a specific cause of ID ^6, 15, 16^.

A special note must be made for the extensive overlap between causes (and pathogenic pathways) of ID and those of autism or ASDs, since many patients have both ID and compromised social interaction and communication and vice versa ^17– 19^. For example, more than 100 genes and 40 genomic loci associated with ASD had been reviewed by Betancur in 2011 ^20^ and all of these were also involved in ID.

## Counting conditions with intellectual disability using OMIM

Curated lists of genes involved in ID have been published by some groups. Gilissen *et al.* ^21^ created two lists including 528 genes with a “confirmed” pathogenetic role and 628 “candidate” genes with mutations reported in fewer than five patients. Another comprehensive list (DDG2P) was prepared to assist the Deciphering Developmental Disorders Study ^22^, including 925 “confirmed” developmental disorder genes up to November 2013 ^23^. Yet another list of 565 genes associated with ID (253 “known” and 312 “candidate”) has been reported by Grozeva *et al.* ^24^, who used the two previous lists as a starting point.

We decided to obtain an independent gene list by using OMIM and the National Center for Biotechnology Information (NCBI) GENE databases. To identify most (if not all) conditions with ID, we searched for entries with either “mental retardation” or “developmental delay”, “intellectual disability”, and “cognitive impairment” in the Clinical Synopsis. It is worth noting that, at least in OMIM, the term “mental retardation” is still the most common (followed by “developmental delay”) term found in the Clinical Synopsis of 981 OMIM entries. Furthermore, only conditions for which at least one gene has been identified were included. This OMIM search resulted in 900 conditions (listed in Supplementary Table 1) and was performed by using the following search string:

((((mental retardation[Clinical Synopsis]) OR developmental delay[Clinical Synopsis]) OR intellectual disability[Clinical Synopsis]) OR cognitive impairment[Clinical Synopsis]) AND “prefix pound”[Properties].

**Figure 3.**
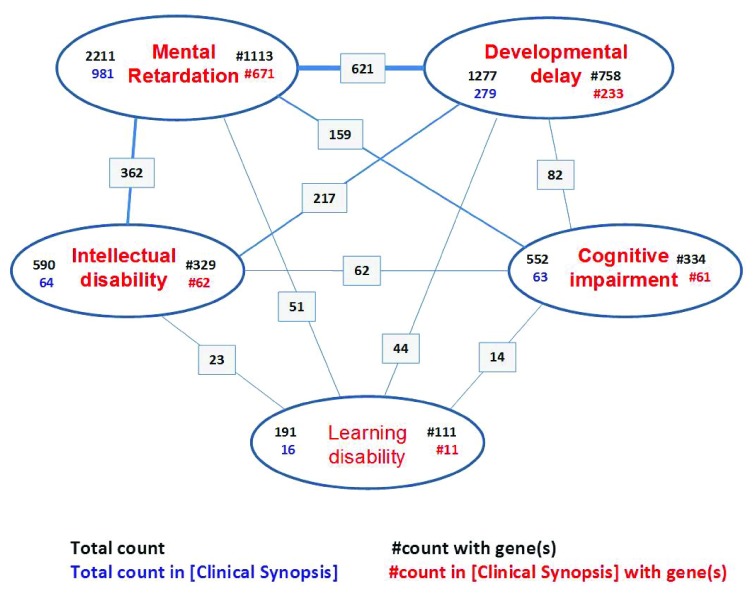
Counts of the major terms describing impaired development of cognitive functions in OMIM. The largest number indicated in black on the upper left of each term is the total number of counts without any limits, whereas the number of entries with the term specified in the Clinical Synopsis is indicated in blue on the lower left. To the right of each term are the counts of entries containing the term linked to at least one gene (upper right with a pound prefix [#], in black) and all entries containing the term in their Clinical Synopsis AND being linked to at least one gene (lower right with a pound prefix [#], in red). OMIM, Online Mendelian Inheritance in Man.

These 900 conditions include several “genomic disorders” (i.e. microdeletion/duplication conditions such as Williams, velo-cardio-facial, and Wolf-Hirschhorn) and even Down syndrome. It is known that a few syndromes associated with these recurrent submicroscopic chromosomal aberrations are actually due to the altered dosage of just one gene ^25– 27^. However, to obtain a list of single genes underlying ID, after transferring the 900 conditions from OMIM to the NCBI GENE database ( Figure 3) and finding 897 items, we manually removed 79 entries without a precise chromosomal location, including those corresponding to genomic disorders (that may be potentially due to more than one gene). This final list contains 818 protein-coding genes and has been ordered either by map_location or by alphabetical order of gene symbol (see Supplementary Table 2). In both lists, removed items are indicated in red.

## Mapping intellectual disability genes and enrichment on the X chromosome

**Figure 4.**
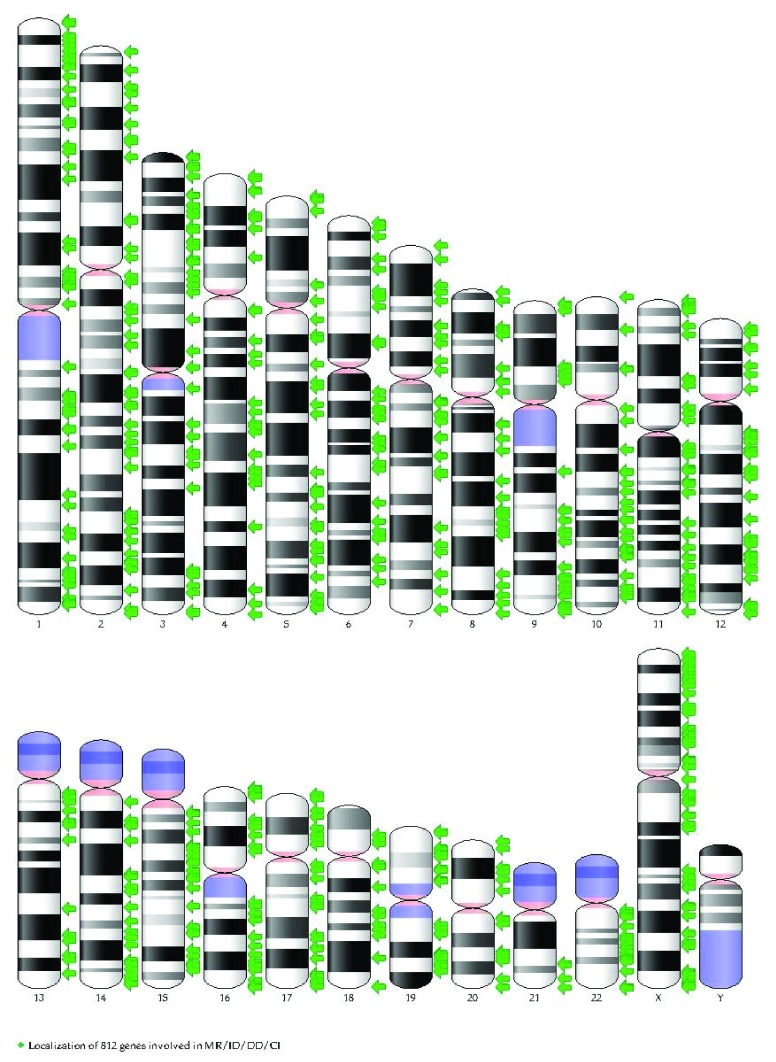
Mapping of the intellectual disability genes on the human karyogram (G-banded) using the Genome Decoration page at National Center for Biotechnology Information. See website at http://www.ncbi.nlm.nih.gov/genome/tools/gdp. Please note that genes causing ID tend to concentrate in G-negative bands, like all other genes. CI, cognitive impairment; DD, developmental delay; ID, intellectual disability; MR, mental retardation.

**Figure 5.**
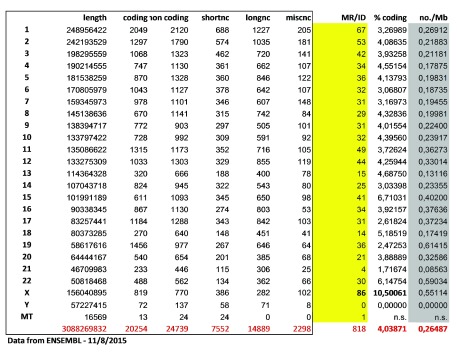
Distribution of intellectual disability genes per chromosome and comparison with the total amount of protein-coding and non-coding genes located on each chromosome. longnc, long non-coding; Mb, megabase; miscnc, miscellaneous non-coding; MR/ID, known mental retardation/intellectual disability genes; shortnc, short non-coding.

We then used the Genome Decoration page at NCBI to map the identified genes on the human karyogram ( Figure 4). Not surprisingly, the density of ID genes is higher in G-negative bands that are typically richer in protein-coding genes. Figure 5 is derived from Supplementary Table 2 and counts the number and proportion of ID genes relative to all protein-coding genes for each individual chromosome. The X chromosome appears to be enriched for genes mutated in patients with ID, be they syndromic or not (10% of all protein-coding genes on the X compared with 4% of the genomic average). Actually, the total number of X-linked ID (XLID) genes is higher than that (86) shown in Figure 5: now (March 2016) the total number of XLID genes is more than 100 out of about 800 protein-coding genes on the X chromosome ^28– 30^. XLID genes have been identified earlier than autosomal ID genes because of their inheritance pattern that allows transmission through several unaffected carrier females, and they may explain part of the reported excess of male patients with ID ^31, 32^. However, is this enrichment real or simply due to ascertainment bias, since the identification of X-linked families is easier? Twenty-five years after the cloning of the first XLMR gene ( *FMR1*, inactivated in the fragile X syndrome), we still do not have a definitive answer to this question and we may have to wait until all ID genes have been identified to settle the dispute. However, several authors suggested the possibility that “intelligence genes” actually concentrated on the X chromosome because of a selective advantage in males ^32– 34^; this evolutionary effect would also explain why intelligence scores appear to be more variable in males compared with females (i.e. males tend to be over-represented at both ends of the general intelligence overall distribution) ^35^.

Over the years, we and others have kept track of XLID conditions and genes ^29, 36– 39^, and both sequencing ^40, 41^ as well as microdeletion/duplication searches ^42^ have been used to identify genetic determinants of XLID. More genes are still being identified with exome sequencing of informative families ^30^.

On the other hand, many syndromes with ID have been linked to autosomal loci, and in recent years a quest for ID genes on the autosomes has progressed rapidly. Recessive forms of both syndromic and “pure” ID have been identified thanks to the study of large consanguineous families coming from non-European countries like Iran ^43, 44^. However, large recessive pedigrees are rare, whereas many sporadic cases are observed among children of non-consanguineous parents, suggesting an autosomal dominant *de novo* origin ^45, 46^. These cases can be diagnosed by using next-generation sequencing (NGS) analysis techniques ^47^ that have become available at more affordable prices in recent years.

Depending on the clinical signs and after an initial screening for fragile X syndrome (mostly with polymerase chain reaction [PCR]-based techniques) and for copy number variants (CNVs), usually with array comparative genomic hybridization (array-CGH), many patients will hopefully receive a diagnosis thanks to NGS using resequencing gene panels, whole exome sequencing (WES), or WGS. Resequencing panels with tens or even hundreds of genes are very useful to screen large cohorts of patients in a cost-effective way and with sufficient confidence to write a report. For example, a diagnostic NGS test screening 99 X-linked and 118 autosomal genes ^48^ has identified a causative mutation in 25% of 96 male and 10 female patients with ID (who had previously tested negative for fragile X and had a normal array-CGH). If WES or WGS is employed and a *de novo* mutation is suspected, it is useful to analyze the proband-parents trio in order to reduce the number of variants. Finally, a note of caution should be made about the interpretation of rare variants: even a *de novo* loss-of-function mutation should not be automatically considered pathogenic, as pointed out by Piton *et al.* ^29^ for some XLID genes.

## Gene Ontology analysis of intellectual disability genes

To provide an overview of the functions of the proteins encoded by genes listed in Supplementary Table 2, we performed a GO analysis using the free tool DAVID (Database for Annotation, Visualization, and Integrated Discovery) 6.7 ( https://david.ncifcrf.gov/) ^49^. We analyzed the 818 official gene symbols with the following DAVID tools: functional clustering, functional annotation, and functional table. To perform the analysis, we selected only the three main GO categories: Biological Process (BP_all), Molecular Function (MF_all), and Cellular Component (CC_all). We used medium stringency and default settings for the analysis, selecting *Homo sapiens* as the background species.

Of the 818 gene symbols, 774 (95%) were present in the DAVID GO dataset. Unmapped IDs are listed in Supplementary Table 3a. The three DAVID functionalities summarize the results in different ways, providing a clustering of the GO terms on the basis of fold enrichment and relationships among ontology terms (functional clustering; see Supplementary Table 3b) or providing statistics of the ontology terms present in the results (functional annotation; see Supplementary Table 3c). The functional chart (see Supplementary Table 3d) reports the GO description for each gene present in the input list. We used functional clustering to highlight the over-represented GO terms, using an arbitrary fold enrichment cutoff of 10.00 (see Supplementary Table 3e). These clusters show an enrichment of cellular organelle (mainly mitochondria) assembly and functions.

**Figure 6.**
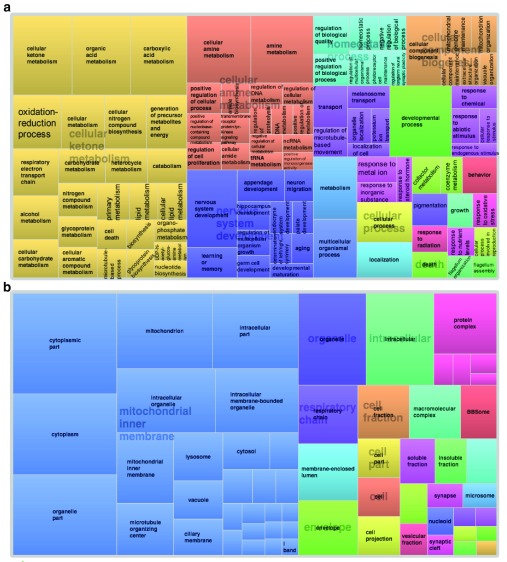
REViGO tree analysis of over-represented Gene Ontology terms obtained with DAVID and associated with the 818 intellectual disability genes. Panel ( **a**) shows Biological Process terms and panel ( **b**) shows Cellular Component terms. DAVID, Database for Annotation, Visualization, and Integrated Discovery; REViGO, Reduce + Visualize Gene Ontology.

GO results, by definition, are redundant and thus can be difficult to visualize. In fact, GO vocabularies are created as acyclic graphs, in which each term follows a hierarchical structure and has a “parent term” and a “child term”, and the complexity is increased by the fact that each term is allowed to have multiple parent and child terms. This confers multiple levels of interpretation to the GO analysis, although the increasing number of parent/child terms does not always add useful information ^50, 51^. To overcome this issue, DAVID functional annotation results, together with their relative *p* values and fold enrichment values (see Supplementary Table 3f), were further used for REViGO (Reduce + Visualize Gene Ontology) analysis ( http://revigo.irb.hr/) ^52^. All terms were included, using the following parameters: allowed similarity = 0.5 (small); first values provided = *p* values; database with GO term sizes = *Homo sapiens*; semantic similarity measure = SimRel. We decided to use the tree view for each main category (Biological Process, Cellular Component, and Molecular Function). Figure 6 shows the two graphs obtained with REViGO summarizing the over-represented (a) Biological Process or (b) Cellular Component GO terms associated with the 818 ID genes. These pictures underline that multiple essential metabolic pathways, especially those related to energy production, are highly associated with the 818 ID genes (a). Also, the Cellular Component GO terms are diversified (b), and mitochondria are well represented.

Finally, we evaluated the 818 ID gene list with g:Profiler ^53, 54^, another useful GO annotation tool ( http://biit.cs.ut.ee/gprofiler/), which scans not only GO terms but also other datasets like the Human Phenotype Ontology project ^55^. Results obtained with g:Profiler are reported in detail in Supplementary Table 4 and confirm the variety of cellular components involved in ID pathogenesis, and mitochondria again show up in the list (see Supplementary Table 4c), and among the top GO Biological Processes are “(central) nervous system development” and “neurogenesis” (see Supplementary Table 4b). Interestingly, when the Human Phenotype Ontology terms are examined (see Supplementary Table 4e), the first two terms of the list (with a highly significant *p* value of 9.61 × 10 ^-297^) are “Neurodevelopmental abnormality” and “Intellectual disability”, followed by “Abnormality of nervous system physiology” ( *p* value: 7.36 × 10 ^-169^) and “Neurodevelopmental delay” ( *p* value: 9.14 × 10 ^-143^).

## Identification of (new) intellectual disability genes

Several strategies have been employed to identify ID genes over the years. A thorough clinical examination of the proband(s) and the reconstruction of the family history are mandatory ^15^ before any attempt is made to pinpoint the responsible gene. In fact, understanding the genetic context (sporadic/familial and dominant/recessive) and collecting all clinical evidence (“diagnostic handles”) facilitate reaching a diagnosis. Furthermore, fragile X syndrome should be excluded by using the available PCR-based tests, considering the frequency of this condition and the dynamic nature of most mutations in the *FMR1* gene ^56^, and array-CGH should be performed as a first-tier test to detect or exclude the presence of potentially relevant CNVs ^57^. It is important to remember that if a CNV is detected, not only is the gene content of the deleted/duplicated region important but also the potential “position effects” (due to deletion or displacement of enhancers) are extremely relevant ^58– 60^. However, even if array-CGH results were normal, a standard karyotype and confirmatory fluorescence *in situ* hybridization (FISH) analysis would still be necessary if a balanced translocation/inversion is suspected.

Then, if clinical examination and the first-tier tests (fragile X and array-CGH) are normal and balanced chromosomal aberrations have been excluded, direct searching for single-nucleotide variants (SNVs) or small insertions/deletions (indels) may be performed by using NGS techniques. Depending on the available resources, including bioinformatic support, either a large panel of known or candidate ID genes can be screened (as shown by 48) or the (currently known) human exome (WES) or genome (WGS) could be investigated. These latter approaches can potentially identify “new genes” responsible for ID, although the number of variants identified in each patient is challenging and not always easily interpreted ^41^. The availability of at least the patient’s parents (trio analysis) facilitates variant interpretation ^45^, and many laboratories prefer to invest the extra resources in order to increase the chances of reaching a diagnosis.

When examining the results of any WES/WGS experiment, known disease genes (e.g. OMIM genes) should be examined first if mutations are identified in any of them, even if the phenotype of proband(s) does not correspond to that already reported in the literature, since phenotypic heterogeneity is common in human genetics. Furthermore, SNV or indels identified in regulatory and untranscribed or untranslated regions of a specific ID gene could eventually be linked to abnormal transcript levels that cause the disease phenotype, as was found to occur in the X-linked *HCFC1* gene ^61^; however, such sequence changes are extremely difficult to detect since they do not fall in the open reading frame and their effect might be appreciated only if mRNA levels were quantitated ^62^. 

In any case, a (long) list of potentially causative variants in several genes is the typical result of WES/WGS experiments and therefore prioritization of candidate variants (based on the presumed effect on the encoded protein) ^63^ is very important to identify the (new) causative ID genes ^64^. Gene prioritization establishes a ranking of candidate genes on the basis of their relevance to the biological process of interest: this is a critical process since the “real” causative gene might be excluded from further analysis depending on the criteria chosen by the researcher. Several computational approaches have been developed for selecting disease candidate genes ^65, 66^ on the basis of either functional (what they do) or topological (where they do it) similarity to known disease genes.

In the postgenomic era, when large sets of data are available on the majority of human genes, numerous correlations can be established to connect genes in networks on the basis of their sequence similarity (paralogues encoding similar proteins), similar transcriptional profile (genes with the same expression in various tissues), similar protein function (GO description), or interaction of the encoded proteins (genes encoding interacting proteins). Systems biology, by integrating heterogeneous datasets such as expression data, sequence information, functional annotation, and the biomedical literature, allows reconstruction of gene networks and molecular pathways relevant for the different physiological and pathological conditions and accelerates the interpretation of monogenic as well as complex neurodevelopmental conditions ^67^.

Very recently, software packages like Exomizer ^68^, PhenIX ^69^, and OVA ^70^ have been made available that also incorporate phenotypic information in the prioritization process, significantly increasing its efficiency ^71^. This extra layer of information, directly related to the specific disease affecting the patient(s), can be added to the bioinformatics analysis pipeline thanks to the terminology standardization efforts of the Human Phenotype Ontology project ^55^.

Finally, given the association between some human diseases and non-coding RNAs ^72^, it is important to keep in mind the possible role of non-coding RNAs in the pathogenesis of ID, as suggested by the analysis performed by Gudenas and Wang ^73^ on long non-coding RNAs and CNVs in ID patients. In fact, pathogenic mutations in RNAs that do not code for proteins shall not be detected by WES and may also be missed by WGS, depending on the quality of sequence annotation.

## Transcriptional profiles, Brain ratio, and Fetal Brain ratio

Probably one of the most relevant factors determining the relevance of a specific gene in causing a given phenotype is its transcriptional profile. When manually inspecting the results of WES/WGS experiments, immediately after scoring for the effect of identified variants on protein sequence, researchers ask about the expression of the candidate gene in the relevant tissue (e.g. brain for the ID phenotype). A number of databases collect mRNA expression data of multiple experiments (for example, the Gene Expression Omnibus [GEO] database, which is available at http://www.ncbi.nlm.nih.gov/geo/). A user-friendly gene expression portal is BioGPS (available at http://biogps.org/), initially established by the Genomics Institute of the Novartis Research Foundation ^74, 75^. Five reference datasets can be visualized with BioGPS, but the most reliable human dataset (GEO dataset GSE1133) explores 79 human tissues—including 21 from the central nervous system (CNS)—and was obtained in 2004 with the Affymetrix U133A arrays ^76^.

We decided to reanalyze the transcriptional profile of 30 brain areas and 49 other tissues of the human body (all in triplicate) that were explored with the Affymetrix U133 Plus 2.0 (a more recent chip with more identified transcripts) by Neurocrine (GEO dataset GSE7307 entitled “Human body index - transcriptional profiling”). Part of this dataset (comprising 20 CNS areas) has been reported by Roth *et al.* ^77^ (2006), but the complete dataset is more comprehensive and gives the opportunity to visualize the transcriptional profile of 20,588 annotated genes and to compare the CNS and the rest of the body. In our analysis, we used the Neurocrine dataset to prioritize all available protein-coding genes on the basis of their relative expression level in the brain ^78^. In fact, since the absolute expression value of a given transcript varies considerably compared with others, we first calculated an average level of expression in both CNS and non-CNS tissues for each available transcript and then we derived a “Brain ratio” (BR) defined as the average expression in (adult) CNS divided by the average expression in all other tissues. Such a ratio allows an easy and efficient comparison between genes with different “absolute” levels of transcription, highlighting those that are relatively more expressed in brain and therefore presumably more important for CNS function (and presumably cognition). We then ranked all 20,588 annotated genes by decreasing BR and found that approximately 8% of all protein-coding genes have a BR above 2 but that approximately 10% of the 818 ID genes and approximately 25% of all XLID genes have a BR above 2 ^78^. Supplementary Table 5 reports the list of the 84 ID genes with a BR of more than 2 (plus two more genes immediately following in the ranking in positions 85 and 86) and their corresponding calculated BRs as well as the functional clustering and annotation obtained with DAVID and the list of GO terms used for REViGO. Finally, we also calculated a “Fetal Brain ratio” (fBR) (expression in fetal brain divided by average expression in adult CNS), and the list of 64 (out of the 818) ID genes with an fBR above 2 is reported in Supplementary Table 6 along with the results of the DAVID analysis.

**Figure 7.**
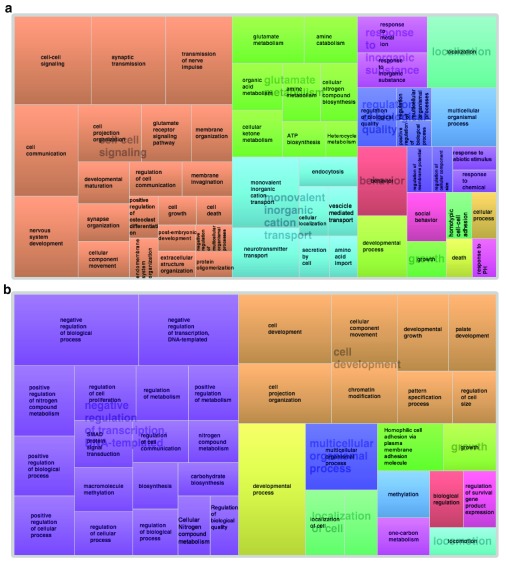
REViGO tree analysis of over-represented Gene Ontology (GO) terms obtained with DAVID. Panel ( **a**) shows GO (Biological Process) terms associated with the 86 intellectual disability (ID) genes with a Brain ratio of 2 or more, while panel ( **b**) shows GO (Biological Process) terms associated with the 64 ID genes with a Fetal Brain ratio of 2 or more. DAVID, Database for Annotation, Visualization, and Integrated Discovery; REViGO, Reduce + Visualize Gene Ontology.

Careful inspection of Supplementary Table 5a suggests that genes with a high BR are usually mutated in nonsyndromic (“pure”) forms of ID but that ID genes with a lower BR (being more ubiquitously expressed) associate with syndromic ID conditions. Similarly, examination of Supplementary Table 6a suggests that genes with a high fBR are sometimes mutated in brain malformations, consistent with their developmental function ^78^. Figure 6 and Figure 7 visually illustrate the above-mentioned concepts thanks to the REViGO analysis of GO terms, and the comparison of the two figures is important: whereas REViGO analysis of all 818 ID genes showed a patchwork of very different Biological Process ( Figure 6a) GO terms, Figure 7a (based on the 84 ID genes with a BR of more than 2) clearly points to cell-cell signaling, synaptic function, and transmission of the nervous impulse and Figure 7b (based on the 64 ID genes with an fBR of more than 2) has 50% of GO terms pointing to regulation of transcription and the other 50% pointing to cell movements and developmental patterning. These differences are also apparent when g:Profiler is used to analyze the GO terms over-represented in these two lists (see Supplementary Table 7).

## Conclusions

To date, more than 800 genes are known to be involved in the pathogenesis of syndromic and nonsyndromic conditions with ID (see Supplementary Table 2), and the functions of their respective proteins are very different. Since 800 out of ~4500 human disease genes currently listed in OMIM is ~18%, if we suppose that the same proportion of all human genes (~20,000) is related to ID, this would suggest that up to 3500 human genes (when mutated) could cause a Mendelian condition that includes ID as one of its components. However, this could be an overestimation since many human morbid genes currently reported by OMIM might have been identified also thanks to their ID phenotype: a more conservative estimate, based on the proportion of 10.5% of all protein-coding genes on the X involved in ID ( Figure 5) that could be extended to the autosomes, leads to an estimate of approximately 2000 genes that, if mutated, would cause syndromic or nonsyndromic ID. Mutations in some of these genes might actually prove lethal during embryogenesis, but thanks to the new powerful sequencing techniques and more sophisticated bioinformatics pipelines, we might eventually identify all remaining protein-coding ID genes.

In any case, analysis of a gene’s transcriptional profile will be useful for the prioritization of candidate genes, and their relative expression in the adult or fetal CNS, estimated with the BR (or fBR), will facilitate comparison among genes with very different absolute levels of transcription. We have to remember that, although we expect that most genes with a high BR (e.g. above 2) will mainly impair cognition whenever mutated, the majority of ID genes are also expressed in many other tissues (i.e. have a low BR) and will usually have a syndromic clinical presentation.

## Supplementary material

**Supplementary Table 1** - OMIM terms search results.

Click here for additional data file..

**Supplementary Table 2** - ID Genes identified through OMIM and NCBI Gene.

Click here for additional data file..

**Supplementary Table 3** - DAVID analysis of ID genes.

Click here for additional data file..

**Supplementary Table 4** - gProfiler analysis of ID genes.

Click here for additional data file..

**Supplementary Table 5** - DAVID and REVIGO analysis of genes with Brain Ratio above 2.

Click here for additional data file..

**Supplementary Table 6** - DAVID and REVIGO analysis of genes with Fetal Brain Ratio above 2.

Click here for additional data file..

**Supplementary Table 7** - gProfiler analysis of genes with Brain/Fetal Brain Ratio above 2.

Click here for additional data file..
